# Diversity, virulence, and antimicrobial resistance of the KPC-producing *Klebsiella pneumoniae* ST307 clone

**DOI:** 10.1099/mgen.0.000110

**Published:** 2017-04-26

**Authors:** Laura Villa, Claudia Feudi, Daniela Fortini, Sylvain Brisse, Virginie Passet, Celestino Bonura, Andrea Endimiani, Caterina Mammina, Ana Maria Ocampo, Judy Natalia Jimenez, Michel Doumith, Neil Woodford, Katie Hopkins, Alessandra Carattoli

**Affiliations:** ^1^​ Istituto Superiore di Sanità, Rome, Italy; ^2^​ Institut Pasteur, Paris, France; ^3^​ University of Palermo, Palermo, Italy; ^4^​ Institute of Infectious Diseases, University of Bern, Bern, Switzerland; ^5^​ Grupo de Microbiología Básica y Aplicada, Escuela de Microbiología Universidad de Antioquia, Medellín, Colombia; ^6^​ Antimicrobial Resistance and Healthcare Associated Infections (AMRHAI) Reference Unit, National Infection Service, Public Health England, London, UK

**Keywords:** KPC, ST307, ST259, plasmid, capsule, WGS

## Abstract

The global spread of *Klebsiella pneumoniae* producing *Klebsiella pneumoniae* carbapenemase (KPC) has been mainly associated with the dissemination of high-risk clones. In the last decade, hospital outbreaks involving KPC-producing *K. pneumoniae* have been predominantly attributed to isolates belonging to clonal group (CG) 258. However, results of recent epidemiological analysis indicate that KPC-producing sequence type (ST) 307, is emerging in different parts of the world and is a candidate to become a prevalent high-risk clone in the near future. Here we show that the ST307 genome encodes genetic features that may provide an advantage in adaptation to the hospital environment and the human host. Sequence analysis revealed novel plasmid-located virulence factors, including a cluster for glycogen synthesis. Glycogen production is considered to be one of the possible adaptive responses to long-term survival and growth in environments outside the host. Chromosomally-encoded virulence traits in the clone comprised fimbriae, an integrative conjugative element carrying the yersiniabactin siderophore, and two different capsular loci. Compared with the ST258 clone, capsulated ST307 isolates showed higher resistance to complement-mediated killing. The acquired genetic features identified in the genome of this new emerging clone may contribute to increased persistence of ST307 in the hospital environment and shed light on its potential epidemiological success.

## Abbreviations

CG, Clonal group; ESBL, Extended-spectrum beta-lactamase; ICE, Integrative conjugative element; IS, Insertion sequence; KPC, Klebsiella pneumoniae carbapenemase; KPC-Kp, KPC-producing Klebsiella pneumoniae; MLST, Multi-locus sequence typing; PBRT, PCR-based replicon typing; ST, Sequence type; T4SS, Type IV secretion system; T6SS, Type VI secretion system; WGS, Whole-genome sequencing.

## Data Summary

1. Whole Genome Shotgun project KP48-IT has been released at DDBJ/ENA/GenBank with the accession number: PRJNA295649 (www.ncbi.nlm.nih.gov/bioproject/295649).

2. Whole Genome Shotgun project PRJNA354908 has been deposited at DBJ/ENA/GenBank with the accession number: PRJNA354908 (www.ncbi.nlm.nih.gov/bioproject/354908), individual accession numbers are listed in Table S1 (available in the online Supplementary Material).

3. Novel plasmid nucleotide sequences have been deposited in GenBank with the accession numbers: pKpQIL_307, KY271403; pKPN3_307_TypeA, KY271404; pKPN3_307_TypeB, KY271405; pKPN307_TypeC, KY271406; pKPN3307_TypeD, KY271407; IncN_TypeA, KY271413; IncN_TypeB, KY271414; IncN_TypeC, KY271415: pTet_ 7201, KY271408.

4. The complete DNA sequences of the following prophage genomes have been deposited in GenBank with the accession numbers: Prophage1_ST307, KY271401; Prophage2_ST307, KY271396; Prophage2b_ST307, KY271395; Prophage3_ST307, KY271397; Prophage4_ST307, KY271398; Prophage5_ST307, KY271399; Prophage6_ST307, KY271400; Phage48_ST307, KY271402.

5. Type A and Type B integrative conjugative elements have been deposited in GenBank with the accession numbers KY271411 and KY271412, respectively.

6. The π-fimbria cluster sequence has been deposited in GenBank with the accession number KY271409.

7. The capsula_entero_ST307 cluster sequence has been deposited in GenBank with the accession number KY271410.

## Impact Statement

*Klebsiella pneumoniae* is one of the most common causes of healthcare-associated infections. The global spread of carbapenemase-producing *Klebsiella pneumoniae* high-risk clones is a public health concern. In the last decade, most hospital outbreaks of carbapenem-resistant *K. pneumoniae* have been attributed to *Klebsiella pneumoniae* carbapenemase (KPC)-producing isolates belonging to clonal group (CG) 258. Recent epidemiological evidence indicates that a new lineage, sequence type (ST) 307 is now emerging, being detected in different parts of the world and outcompeting the international CG258 in some hospital settings. Here we report the first description, to our knowledge, of ST307 genomes, from isolates of different geographical origins. In particular, we characterized the resistome and mobilome of these genomes, which comprised a variety of resistance and virulence determinants located on plasmids, integrative conjugative elements, and phages. Resistance to human sera of this newly-emerging clone was also measured. Some of the genetic features of ST307 described in this study are novel or rarely reported in species of the genus *Klebsiella* and may help in tracing the emergence of ST307 isolates in future surveillance studies performed around the world.

## Introduction

The worldwide spread of carbapenemase-producing *Klebsiella pneumoniae* (*Kp*) has become a major threat for healthcare facilities [1]. This global phenomenon has been mainly associated with the clonal dissemination of high-risk clones. One of the most succesful is the *Klebsiella pneumoniae* carbapenemase (KPC)-producing *Kp* (KPC-*Kp*) sequence type (ST) 258 clone, and its related variants belonging to clonal group 258 (CG258) [2, 3]. However in recent years, new extensively drug-resistant lineages have emerged internationally [1, 4–8]. Among them, KPC*-Kp* ST307 is a candidate for becoming one of the most clinically relevant clones, since its emergence has been recognized in several countries in the last five years [9–12]. ST307 was first defined in 2008 in the multi locus sequence typing (MLST) database (an unpublished isolate), and has since been described in 2013 in the USA [10]. It can be proposed that ST307 was initially associated with the production of the globally disseminated extended-spectrum beta-lactamase (ESBL) CTX-M-15. The acquisition of a KPC enzyme was subsequent to that of CTX-M-15, as deduced from the fact that CTX-M-15-producing *Kp* ST307 have been previously reported at high frequencies in Italy, Korea, Pakistan and Morocco and in pets from Japan [9–15].

The spread of ST307 strains in Italy is particularly interesting because it traces the evolution of this particular clone over time, starting with its introduction by replacement among KPC producers of CG258. The first outbreak of KPC-3-producing *Kp* ST258, in Palermo, Sicily, occurred in 2008 [16], and then this clone became a key epidemiological feature in many healthcare facilities of the region until 2013 [17]. Results of a surveillance study performed in March–August 2014 in the three largest hospitals of Palermo revealed a change in epidemiology, with multifocal dissemination of KPC-3-producing *Kp* clones observed. In particular, the predominant KPC-3 CG258 clone was identified in 38 out of 94 (40 %) patients, but 27 out of 94 (28 %) isolates were ST307 producing both KPC-3 and CTX-M-15 [18, 19]. In Colombia, the results of a two-year surveillance study performed on 193 carbapenem-resistant *Kp* strains collected between June 2012 and June 2014 in five tertiary-care centres in Medellín indicated that 62.2 % of the isolates were from STs unrelated to CG258, of which 14.2 % were ST307 [20]. Patients infected with KPC-*Kp* ST307 presented high mortality (over 50 %) and had a longer hospital stay compared with patients infected with other clones, indicating that this lineage encodes additional factors contributing to its virulence. The prevalence of ST307 in the UK remains relatively low, as observed by the National Infection Service, Public Health England in the United Kingdom with only eight KPC-*Kp* ST307 isolates identified in a collection of over 1600 carbapenemase-producing *Kp* isolates sequenced from 2014 to 2016. Interestingly, one of these isolates originated from a patient transferred to the UK from Italy.

In this work, we performed whole-genome sequencing (WGS) and compared the genetic structures of KPC-*Kp* ST307 isolates from Italy, Colombia and the UK in order to identify factors that could contribute to the success and spread of this newly emerging clone.

## Methods

### Clinical isolates and sequenced strains

A total of 24 ST307 KPC-*Kp* isolates were studied (Table 1). Half of the isolates (*n*=12) were selected from 27 ST307 KPC-3-*Kp* isolates collected during the surveillance study performed in March–August 2014 in Palermo [18]; they were representatives of the isolates obtained from the three participating hospitals, and were selected based on slightly different pulsed-field gel electrophoresis (PFGE) patterns identified in the survey (defined as D1 to D4 subtypes) [18, 19]. Among these 12 Italian strains, only seven were CTX-M-15 producers (Table 1). The remaining isolates included four Colombian isolates carrying genes encoding KPC-2 (*n*=2) or KPC-3 (*n*=2) from two different hospitals that were selected from the collection of 17 KPC-ST307 *Kp* obtained during a two-year surveillance study [20] and eight KPC-*Kp* isolates from the UK, including the only CG307 KPC-*Kp* identified among over 3000 Carbapenem Producer Enterobacteriaceae sequenced by the National Infection Service, Public Health England [Table 1; Antimicrobial Resistance and Healthcare Associated Infections (AMRHAI) Reference Unit, unpublished data]. Seven of the UK isolates belonged to ST307 and one was a single-locus variant of ST307 (H154440769-UK). Interestingly, one of the UK strains (strain H155360912-IT) was isolated from a patient who had been transferred from Italy.

**Table 1.** Characteristics and features of the ST307 K. pneumoniae isolates from the three countries Whole-genome sequencing was performed for strains indicated with underlined type.

Strain	Country	Carbapenemase and ESBL		Plasmids						Capsule		π-fimbria
				Replicons	Urea	Glycogen	pKPN307 type	pKpQIL KPC-3	IncN KPC-2 type	Cp1 wzi	Cp2	
48-IT	IT	KPC-3	CTX-M-15	FIIK, FIBK	+	+	A	+	−	173	+	+
CIV2-IT	IT	KPC-3	CTX-M-15	FIIK, FIBK	+	+	A	+	−	173	+	+
CIV10-IT	IT	KPC-3	CTX-M-15	FIIK, FIBK	+	+	A	+	−	173	+	+
VSC1-IT	IT	KPC-3	CTX-M-15	FIIK, FIBK	+	+	A	+	−	173	+	+
CIV13-IT	IT	KPC-3	CTX-M-15	FIIK, FIBK	+	+	A	+	−	173	+	+
CIV66-IT	IT	KPC-3	CTX-M-15	FIIK, FIBK	+	−	B	+	−	173	+	+
CIV65-IT	IT	KPC-3	CTX-M-15	FIIK, FIBK	+	−	B	+	−	173	+	+
KH-24-CO	CO	KPC-3	CTX-M-15	FIIK, FIBK	+	+	A	−	−	173	+	+
KH-37-CO	CO	KPC-3	CTX-M-15	FIIK, FIBK	+	+	A	−	−	173	+	+
KH-43-CO	CO	KPC-2	CTX-M-15	N, FIIK, FIBK	+	+	A	−	−	173	+	+
KL-49-CO	CO	KPC-2	CTX-M-15	N, FIIK, FIBK	+	+	A	−	A	173	+	+
H151300628-UK	UK	KPC-2	CTX-M-15	N, FIIK, FIBK	+	−	B	−	B	173	+	+
H151400610-UK	UK	KPC-2	CTX-M-15	N, FIIK, FIBK	+	−	B	−	B	173	+	+
H151400611-UK	UK	KPC-2	CTX-M-15	N, FIIK, FIBK, FIBM, HIBM	+	−	B	−	C	173	+	+
H151440672-UK	UK	KPC-2	CTX-M-15	N, FIIK, FIBK	+	−	B	−	B	173	+	+
H154440769-UK	UK	KPC-2	CTX-M-15	FIIK, FIBK	+	+	A	+	−	173	+	+
H150820806-UK	UK	KPC-2	−	FIIK, FIBK	+	+	C	+	−	173	+	+
CIV57-IT	IT	KPC-3	−	FIIK, FIBK	−	−	D	+	−	173	+	+
H155360912-IT	UK	KPC-3	−	FIIK, FIBK, R	−	−	−	+	−	173	+	+
H151440671-UK	UK	KPC-2	−	N	−	−	−	−	B	173	+	+
CIV4-IT	IT	KPC-3	−	FIIK, FIBK	−	−	D	+	−	−	+	+
CIV78-IT	IT	KPC-3	−	FIIK, FIBK	+	+	C	+	−	−	+	+
21-IT	IT	KPC-3	−	FIIK, FIBK	+	+	C	+	−	−	+	+
VSC21-IT	IT	KPC-3	−	FIIK, FIBK	+	−	B	+	−	−	+	+

In addition to the eight sequenced isolates from the UK, the complete genome sequences of four additional strains were obtained: isolates 48-IT and CIV4-IT were chosen for WGS from the Italian collection as representatives of the KPC-3- and CTX-M-15-positive, and KPC-3-positive, CTX-M-15-negative isolates, respectively. KL-49-CO and KH-43-CO were chosen for WGS as representatives of the KPC-2-positive strains from Colombia.

### Whole-genome sequencing

Genomic DNA was purified from strain 48-IT using a DNA extraction kit (Macherey Nagel). Plasmid DNA from 48-IT was purified using a Plasmid Midi Kit (Invitrogen). Genomic and plasmid DNA were used to prepare two different shotgun libraries, which were sequenced on the 454-GS platform following the standard sequencing procedure (Roche Diagnostics). Reads obtained were assembled using the GS-FLX gsAssembler software (Roche Diagnostics).

WGS of CIV4-IT, KH-43-CO and KL-49-CO was performed on DNA extracted using the Macherey Nagel kit. Genomic DNA paired-end libraries were generated using the Nextera XT DNA sample preparation kit (Illumina) and sequenced using a MiSeq instrument with 2×300 PE protocol (Illumina).

DNA from UK isolates was extracted with the Qiasymphony DSP (Qiagen). DNA libraries were prepared using the Nextera XT sample preparation method and sequenced with a standard 2×100 PE protocol on a HiSeq 2500 instrument (Illumina). *De novo* assembly of Illumina reads was performed using the Galaxy version 20150522 of A5 pipeline through the ARIES public Galaxy server (https://w3.iss.it/site/aries/) [21].

### Genome annotation

Draft genome sequences were annotated using the RAST server (http://rast.nmpdr.org/) and BASYs Server [22].

Antimicrobial resistance and replicon genes were detected using the ResFinder [23] and PlasmidFinder [24] online tools (https://cge.cbs.dtu.dk/services/).

Virulence genes were identified using the BIGSdb‐Kp database, at the Institut Pasteur (http://bigsdb.pasteur.fr/klebsiella/klebsiella.html). Phage sequences were identified using the PHAST prediction tool (http://phast.wishartlab.com/). Capsular types were deduced by sequence comparison of the *wzi* genes with those previously described [25].

### Genome comparative analysis

Genomes were compared using the SEED Viewer version 2.0 (http://rast.nmpdr.org/seedviewer.cgi) and MAUVE comparison tools. The 48-IT strain was used as the reference genome for comparison (i) with the other sequenced ST307 genomes in order to identify genetic variations occurring within the clone (Fig. S1) and (ii) with the ST258 *Kp* NJST258_2 (CP006918), ST258 KPNIH1 (CP008827) and ST11 HS11286 (CP003200) reference genomes [26–28]. Genomic differences identified between the 48-IT strain and reference genomes were confirmed among the three other sequenced ST307 strains (Figs S2 and S3).

Major genomic differences, defined by the presence or absence (<70 % amino acid identity) of at least five consecutive coding sequences (Table S2), were manually curated and the respective DNA sequences of these regions were further characterized by blastn (http://blast.ncbi.nlm.nih.gov/Blast.cgi) against the NCBI database.

Core genome MLST was performed using the BIGSdb tool [29] built in the Institut Pasteur database (http://bigsdb.pasteur.fr). Genetic relatedness was assessed by the neighbor-joining method from a distance matrix consisting of pairwise differences between the allelic profiles of 634 genes constituting the strict core-genome of *K. pneumoniae* as defined in the MLST scheme [4, 30].

The integration site of integrative conjugative elements (ICEs) was determined by comparing *in silico* the 48-IT and H151440671-UK sequenced genomes with the genome of the *K. pneumoniae* strain CAV1193 (CP013322) [31] sequences known to lack the yersiniabactin ICE. ICE groups and tRNA-Asn integration sites followed the classification proposed by Marcoleta *et al*. [32].

### Plasmid reconstruction

Plasmid DNA was purified from strain 48-IT with a Plasmid Midi Kit (Invitrogen) and used to transform *Escherichia coli* DH5α chemically competent cells (Invitrogen). Transformants were selected on Luria–Bertani agar plates (Sigma), containing ampicillin (50 µg ml^−1^), and checked for *bla*_KPC-3_-positives by PCR.

Plasmid contigs were first assembled using the 454 ReadStatus output file, generated by the gsAssembler software (Roche Diagnostics), and then ordered by identifying reads overlapping adjacent contigs. Plasmid pKpQIL-like carrying *bla*_KPC-3,_ originally assembled in nine contigs, was reconstructed using a PCR-based gap closure method with the 48-IT transformant DNA as the template. The fully reconstructed plasmid, named pKpQIL-307 was submitted to GenBank as the prototype of this plasmid type.

Another 30 contigs showing similarities to a pKPN3-like plasmid were also identified from 48-IT plasmid sequences. The complete sequence was reconstructed by PCR to close all the gaps, and the resulting plasmid, named pKPN-307 type A, was submitted to GenBank as the prototype of this plasmid type. In addition to strain 48-IT, contigs showing 99 % sequence identity and 100 % coverage with pKPN-307 were also identified in genomes of H154440769-UK (43 contigs), KH-43-CO (20 contigs) and KL-49-CO (19 contigs).

In the other sequenced ST307 genomes, plasmid contigs were identified using ResFinder and PlasmidFinder and by blastn against the pKPN-307, pKpQIL-307 and R46 IncN reference plasmids. The order and orientation of plasmid contigs established by alignment to the three reference plasmids were verified by checking overlapping paired-end reads, and, for some prototypes, confirmed by a PCR-based gap closure method.

In detail, plasmid pKPN-307 type B was obtained from H151440672-UK (21 contigs, considered as the prototype of pKPN-307 type B and submitted to GenBank), H151300628-UK (22 contigs), H151400611-UK (20 contigs) and H151440672-UK (21 contigs). Plasmids pKPN-307 type C and D were obtained from H150820806-UK (62 contigs) and CIV4-IT (23 contigs), respectively.

Plasmids IncN type A and C were fully assembled from the genomes of KL-49-CO (six contigs) and H151400611-UK (ten contigs), respectively. Plasmid IncN type B was assembled from H151440671-UK (eight contigs, considered as the prototype of IncN type B and submitted to GenBank), H151300628-UK (12 contigs), H151400610-UK (11 contigs) and H151440672-UK (15 contigs).

Plasmids pTetA and pTetA-QnrB1 were initially assembled in one contig with complementary paired-ends from the genomes of H151440672-UK and KH-43-CO, respectively. Manual annotation of complete plasmid sequences was performed using Artemis Version 8 (Sanger Institute) in combination with blastp homology searches (http://blast.ncbi.nlm.nih.gov/Blast.cgi).

### Serum resistance

Serum resistance assays were performed by mixing 100 µl of *K. pneumoniae* overnight bacterial LB liquid culture diluted to a final concentration of 1.5×10^4^ cells ml^−1^ with a pool of 300 µl of fresh, non-heated human sera obtained from three healthy volunteers. The 1 : 3 bacteria : sera volume ratio mixture was incubated at 37 °C and aliquots of 100 µl at T_0_ and after 30, 60 and 120 min were plated on LB agar and incubated overnight at 37 °C prior to viable cell counts. The assays were repeated three times using three different pools of sera obtained from different volunteers.

### PCR analysis of ST307 specific features

Specific features identified in the ST307 genomes were screened for by PCR in the entire collection of isolates listed in Table 1, using the primer pairs detailed in Table S3 with total DNAs extracted with a Macherey Nagel kit. PCRs were performed using the following conditions: one cycle of denaturation at 94 °C for 5 min, followed by 30 cycles of denaturation at 94 °C for 1 min, annealing temperature as defined in Table S3 for each set of primers for 30 s and elongation at 72 °C for 1 min and a final extension cycle at 72 °C for 5 min. The *bla*_CTX-M_ and *wzi* genes were screened for by PCR as previously described [25, 33] and the amplified products were fully sequenced.

Plasmid typing was performed using the PCR-Based Replicon Typing Kit (PBRT-KIT, DIATHEVA).

## Results

### Whole-genome sequencing of KPC-*Kp* ST307

A total of 24 ST307 isolates from Italy, Colombia and the UK were studied, of which 12 isolates, representative of our collection were sequenced (Table S1).

**Fig. 1.**
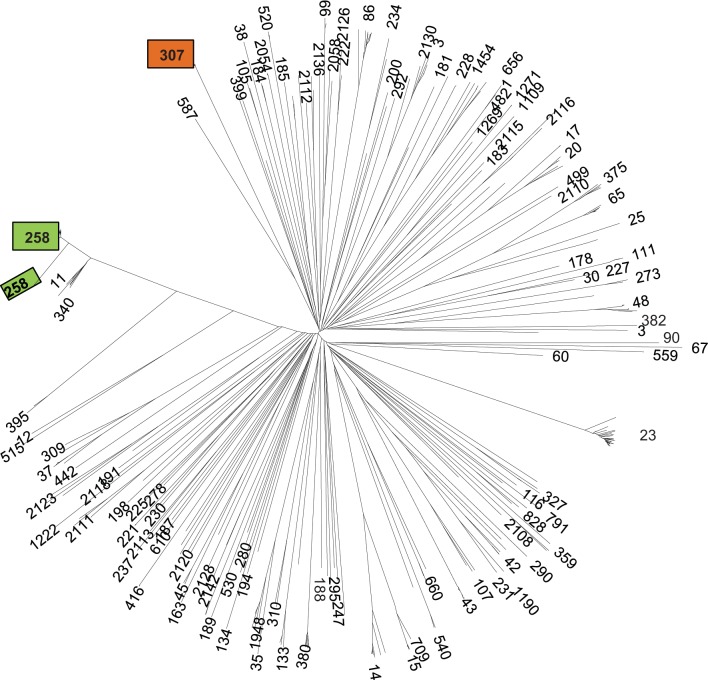
Unrooted neighbor-joining tree of *K. pneumoniae* core genome. The tree is based on the proportion of mismatches among allelic profiles of the strict core genome MLST scheme. Numbers at the tips of branches are sequence types. The positions of ST307 and the two ST258 clades are indicated by orange and green boxes, respectively.

Cluster analysis based on 634 strict core-genome MLST genes demonstrated the clear phylogenetic distinction of the ST307 genomes from those of previously analyzed isolates, indicating that they represent a unique sub-lineage (or clonal group) of *K. pneumoniae* very distant from the two ST258 clades, but not unusually divergent from other *K. pneumoniae* (Fig. 1).

### WGS comparative analysis

Comparative analysis of strains from Italy, Colombia and the UK demonstrated that the major differences in the genomes of ST307 were in antimicrobial resistance gene complement (resistome) and plasmid and phage gene content (mobilome).

The ST307 core genome was highly conserved among strains, while different variants of plasmids and ICEs were detected (Fig. S1). A total of 4745 common genes (cut-off, over 95 % amino acid identity) and 637 accessory genes, present in at least one of the 12 sequenced isolates, were identified. Among accessory genes, 202 were hypothetical proteins with unknown function and 83 were phage-associated proteins.

Comparison of ST307 48-IT with ST258 NJST258_2 (CP006918), ST258 KPNIH1 (CP008827) and ST11 HS11286 (CP003200) genomes, using the Seed Viewer (blastp-based comparison, Fig. S2) and MAUVE alignment tools (blastn-based alignment, Fig. S3), identified 15 major regions of discontinuity (Table S2). Regions that were unique to 48-IT, being absent in at least one of the other three reference genomes but present in all the ST307 genomes, encoded capsules, LPS modification systems, fimbriae, secretion and efflux systems.

### ST307 resistome

Among the analysed strains 16, including 4 out of 4 Colombian, 7 out of 12 Italian and 5 out of 8 UK strains, were CTX-M-15-positive among which nine were KPC-3 producers and seven were KPC-2 producers (Table 1). Overall, the *bla*_KPC-3_ gene was detected in 12 out of 12 Italian, 2 out of 4 Colombian and 1 out of 8 UK isolates (the latter imported from Italy). The *bla*_KPC-2_ gene was detected in all remaining isolates, including seven from the UK and two from Colombia. Most of the strains carried additional acquired resistance genes, such as *bla*_TEM_, *bla*_OXA_, aac(3)-IIa, *aac*(*6′*)*Ib-cr*, *qnrB*, *tet*(A), *strAB*, *sul2*, *dfrA14* and *catB3*, but the complement of resistance genes differed among the isolates. The *bla*_SHV-28_, *oqxAB* and *fosA* genes, previously described as intrinsic to *K. pneumoniae*, were detected in all genomes (Table 2).

**Table 2.** Resistome, integrative conjugative elements, prophages and phages in ST307 genomes

Strain	Beta-lactamases	Aminoglycosides	Quinolones	Others	ICE-YB	Prophages						Phage
48-IT	*bla*_KPC-3_, *bla*_CTX-M-15_, *bla*_TEM-1B_, *bla*_OXA-1_, *bla*_OXA-9_, *bla*_SHV-28_	*strA*, *strB*, *aac*(*3*)*-IIa*, *aac*(*6* *′*)*Ib-cr*	*qnrB1*, *oqxAB*	*sul2*, *dfrA14*, *catB3*, *fosA*	A	Ф1	Ф2c					Ф48
CIV4-IT	*bla*_KPC-3_, *bla*_TEM-1A_, *bla*_OXA-1_, *bla*_OXA-9_, *bla*_SHV-28_	*aac*(*6′*)*Ib-cr*	*qnrB1*, *oqxAB*	*dfrA14*, *catB3*, *fosA*	A	Ф1	Ф2c					
KH-43-CO	*bla*_KPC-2_, *bla*_CTX-M-15_, *bla*_TEM-1B_, *bla*_OXA-1_, *bla*_SHV-28_	*strA*, *strB*, *aac*(*3*)*-IIa, aac*(*6′*)*Ib-cr*	*qnrB* *1*, *oqxAB*	*sul2*, *dfrA14*, *tetA*, *catB3*, *fosA*	−	Ф1	Ф2b		Ф4	Ф5	Ф6	
KL-49-CO	*bla*_KPC-2_, *bla*_CTX-M-15_, *bla*_TEM-1B_, *bla*_OXA-1_, *bla*_SHV-28_	*strA*, *strB*, *aac*(*3*)*-IIa*, *aac*(*6′*)*Ib-cr*	*qnrB1*, *oqxAB*	*sul1*, *sul2*, *dfrA14*, *tetA*, *catB3*, *fosA*	−	Ф1	Ф2b	Ф3	Ф4			
H150820806-UK	*bla*_KPC-2_, *bla*_TEM-1A_, *bla*_OXA-1_, *bla*_OXA-9_, *bla*_SHV-28_	*aac*(*6′*)*Ib-cr*	*qnrB1*, *oqxAB*	*dfrA14*, *tetA*, *catB3*, *fosA*	−	Ф1		Ф3	Ф4			
H154440769-UK	*bla*_KPC-2_, *bla*_CTX-M-15_, *bla*_TEM-1B_, *bla*_OXA-1_, *bla*_OXA-9_, *bla*_SHV-28_	*strA*, *strB*, *aac*(*3*)*-IIa*, *aac*(*6′*)*Ib-cr*	*qnrB1*, *oqxAB*	*sul2*, *dfrA14*, *tetA*, *catB3*, *fosA*	−	Ф1						
H151300628-UK	*bla*_KPC-2_, *bla*_CTX-M-15_, *bla*_TEM-1B_, *bla*_OXA-1_, *bla*_SHV-28_	*strA*, *strB*, *aac*(*3*)*-IIa*, *aac*(*6′*)*Ib-cr*	*qnrB1*, *oqxAB*	*sul2*, *dfrA14*, *tetA*, *fosA*	B	Ф1	Ф2					
H151400610-UK	*bla*_KPC-2_, *bla*_CTX-M-15_, *bla*_TEM-1B_, *bla*_OXA-1_, *bla*_SHV-28_	*strA*, *strB*, *aac*(*3*)*-IIa*, *aac*(*6′*)*Ib-cr*	*qnrB1*, *oqxAB*	*sul2*, *dfrA14*, *tetA*, *fosA*	B	Ф1	Ф2					
H151400611-UK	*bla*_KPC-2_, *bla*_CTX-M-15_, *bla*_TEM-1B_, *bla*_OXA-1_, *bla*_SHV-28_	*strA*, *strB*, *aac*(*3*)*-IIa*, *aac*(*6′*)*Ib-cr*	*qnrB1*, *oqxAB*	*sul2*, *dfrA14*, *tetA*, *fosA*	B	Ф1	Ф2					
H151440672-UK	*bla*_KPC-2_, *bla*_CTX-M-15_, *bla*_TEM-1B_, *bla*_OXA-1_, *bla*_SHV-28_	*strA*, *strB*, *aac(3)-IIa*, *aac*(*6′*)*Ib-cr*	*qnrB1*, *oqxAB*	*sul2*, *dfrA14*, *tetA*, *fosA*	B	Ф1	Ф2					
H155360912-IT	*bla*_KPC-3_, *bla*_TEM-1A_, *bla*_OXA-9_, *bla*_SHV-28_		*oqxAB*	*fosA*	B		Ф2					
H151440671-UK	*bla*_KPC-2_, *bla*_TEM-1B_, *bla*_SHV-28_		*oqxAB*	*fosA*	B	Ф1	Ф2					

### ST307 mobilome – plasmids

Plasmids from the 12 ST307 genomes were classified into four major groups based on replicon and resistance gene content:

#### The KPC-carrying plasmids

**Fig. 2.**
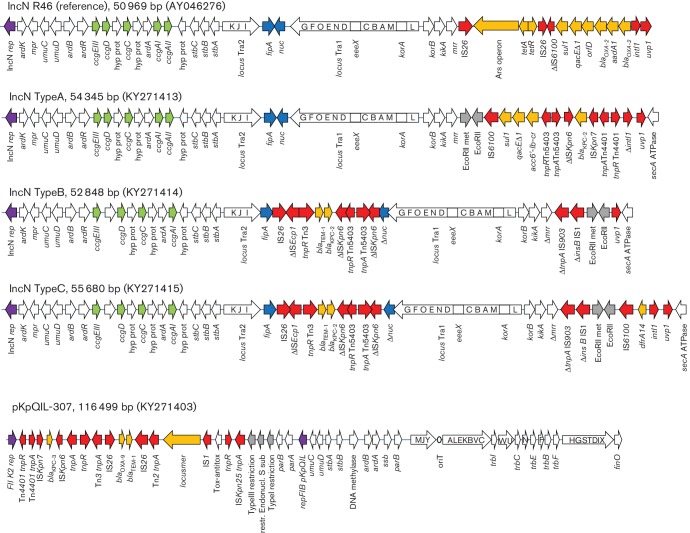
KPC-positive plasmids identified in ST307. White arrows indicate plasmid scaffold genes and their direction of transcription. The locus Tra is indicated by a squared white arrow with capital letters indicating the respective *tra* genes (i.e.: *traG*, G; *traF*, F; *traO*, O etc.). Resistance genes are indicated by orange arrows. Transposon-related genes (*tnpA*, tnpR and *tnpM*], class 1 integrase and insertion sequences are indicated by red arrows. Other genes are indicated by coloured arrows as follows: violet, replicase genes; grey, restriction enzyme and DNA methylase genes; green, *ccg* cluster; blue, *fipA* and *nuc* genes.

The *bla*_KPC-3_ gene was always located on pKpQIL-like plasmids, 1 16 499 bp in size, which were highly similar to those previously described in CG258 [2, 34] and characterized by the presence of two replicons (FIIk2 and FIB-pKpQIL). In contrast, the *bla*_KPC-2_ gene was located on three different plasmid structures: pKpQIL-like, IncN and untypable (in the KH-43-CO strain) plasmids. In total, three types of IncN plasmids, named A, B and C, were identified [35]. Type A was detected in a single Colombian isolate while types B and C were found in isolates from the UK (Table 1, Fig. 2).

IncN type A was 54 345 bp in size, showed integration of the Tn*5403*-ΔIS*Kpn6- bla*_KPC-2_–IS*Kpn7* transposon into a class 1 integron and contained the *aac*(*6′*)*Ib-cr* gene cassette located close to the *uvp1* resolvase gene.

IncN type B was 52 848 bp in size and showed integration of a composite *bla*_KPC-2_ gene environment including IS*26*, ΔIS*Ecp1*, a portion of the Tn*2-bla*_TEM-1_ transposon and the deleted ΔIS*Kpn6* element of Tn*4401* integrated within the coding sequence of the *nuc* gene.

IncN type C was 55 680 bp in size and was identical to type B except that it carried a Δ3′CS-class 1 integron carrying the *dfrA14* gene cassette close to the *uvp1* resolvase gene.

#### The pKPN-307 plasmids

**Fig. 3.**
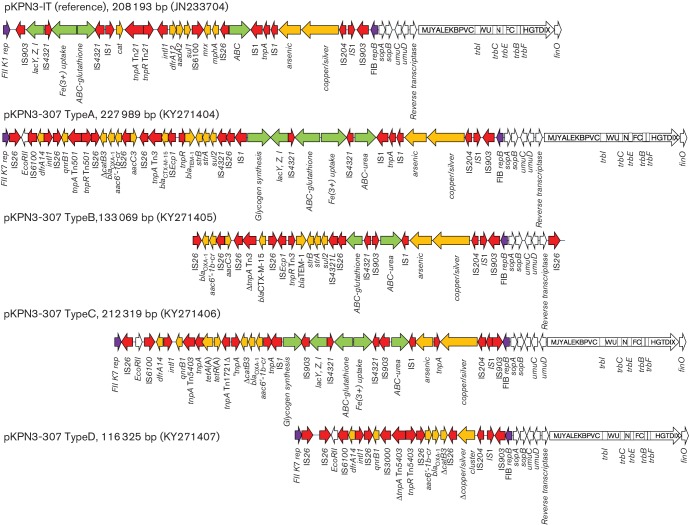
Variant pKPN-307 plasmids identified in ST307. White arrows indicate plasmid scaffold genes and their direction of transcription. The locus Tra is indicated by a squared white arrow with capital letters indicating the respective *tra* genes (i.e.: *traG*, G; *traF*, F; *traO*, O etc.). Resistance genes are indicated by orange arrows. Transposon-related genes (*tnpA*, *tnpR*, *tnpM*), class 1 integrase and insertion sequences are indicated by red arrows. Other genes are indicated by coloured arrows as follows: violet, replicase genes; green, clusters encoding putative virulence determinants.

Four variants of pKPN-307 were identified and named type A to D (Table 1, Fig. 3).

Type A pKPN-307 was 2 27 989 bp in size, carried two replicons (FIIk7 and FIB-pKPN3) and a multi-drug resistance region (MDR) of 38 kb comprising the *bla*_CTX-M-15_, *bla*_TEM-1B_, *bla*_OXA-1_, *aac*(*3*)*-IIa*, *aac*(*6′*)*Ib-cr*, *qnrB1*, *strAB*, *sul2*, *dfrA14* and partial *catB3* genes and the arsenic, copper and silver resistance clusters. Five putative virulence-encoding clusters were identified: the *lac* operon, the Fec-like iron (III) dicitrate transport system, a glutathione ABC-transport system [36], a novel urea ABC-transport system and a novel cluster for glycogen synthesis. The urea transport system included UrtA (urea binding protein), UrtB (urea permease), UrtC (urea transporter) and AmiF (formamidase). The glycogen synthesis cluster included a 4-α-glucan branching enzyme, glucose-1-phosphate adenylltransferase, glycogen synthase, glycogen phosphorylase and phosphoglucomutase enzymes.

Type B pKPN-307 was 1 33 069 bp in size, carried the FIB-pKPN3 replicon, arsenic and copper/silver resistance clusters and the same MDR region as found in type A plasmids, but lacked *catB3*, *qnrB* and *dfrA14* genes, and the FIIk7 replicon and transfer locus. This plasmid carried a urea ABC-transport system and a partial glutathione ABC-transport system.

Type C pKPN-307 was 2 12 319 bp in size. In this plasmid the virulence clusters, transfer and arsenic and copper/silver resistance loci, and replicons were as found in type A plasmids, while the MDR region lacked the *bla*_CTX-M-15_, *aac*(*3*)*-IIa*, *strAB* and *sul2* resistance determinants.

Type D pKPN-307 was 1 16 325 bp in size. Transfer region and replicons were as in type A plasmids but the MDR region carried only the *bla*_OXA-1_, *aac*(*6′*)*Ib-cr*, *qnrB1* and *dfrA14* genes, and a partial copper/silver resistance determinant. None of the five virulence clusters identified in type A plasmids were detected on this plasmid variant.

#### The pTet plasmids

The Tn*1721 *::* tet*(A) element was identified on approximately 5 kb plasmids in two strains from the UK and in KL-49-CO from Colombia. In strain KH-43-CO a *tet*(A)–*qnrB1* plasmid of 13 262 bp was detected.

#### Other large plasmids

FIB-M, HIB-M and R replicons were identified in the genomes of two UK isolates (Table 1). It was not possible to get complete assembly of plasmids carrying these replicons and to link them to any resistance gene(s) because of the short contigs generated by short-read WGS technology and the lack of a proper reference plasmid for the assembly.

The remaining 12 ST307 isolates that were not sequenced were screened by PCR to detect the most relevant plasmid-mediated features identified (Table 1). Overall the PCR results confirmed the frequent association of pKpQIL with KPC-3 and IncN plasmid with KPC-2. CTX-M-15 was associated with the pKPN-307 plasmids. Type A pKPN-307 was the most diffused pKPN-307-like plasmid, being present in strains from Italy, Colombia and the UK; pKPN-307 types B and C were detected in Italian and UK isolates, whereas pKPN-307 type D was present only in two Italian isolates (CIV57-IT and CIV4-IT).

### ST307 mobilome – prophages, phages and integrative conjugative elements

Six different prophages were identified in the ST307 genomes, but φ1 and φ2 were more prevalent, being detected in 9 out of 12 genomes. One extrachromosomal phage was identified in strain 48-IT (Table 2, Figs S1 and S4).

ICEs associated with the cluster encoding the yersiniabactin virulence trait [37, 38] were found in 8 out of 12 genomes (Table 2) but had two different structures designated ICE-YB-Type A and B. Both ICE types were constituted of a Type IV secretion system (T4SS) and carried the *mobA* and *mobB* genes but had different *ybt*, *irp1*, *irp2* and *fyuA* alleles (Table S4) and showed a different assortment of associated ORFs encoding conserved or hypothetical proteins. Compared with published sequences, the ICE-YB-Type A shared 99 % identity and 96 % coverage with the group VI ICE previously described in the HS11286 reference genome [32]. Type B showed 98 % sequence identity and 86 % coverage with the same group VI ICE, but included an additional 8 kb region, encoding restriction–﻿methylation enzymes, an ABC transport system and hypothetical proteins. Among the hundreds of genomic sequences available in public databases, only four matches (SKGH01-ST147, CP015500.1; CAV1016-ST45, CP017934.1; RJF293-ST374, CP014008.1; *E. coli* ED1a, CU928162.2) showing identity with this 8 kb terminal portion of the ICE were identified by blastn, indicating that this is a novel and rare type of ICE element.

**Fig. 4.**
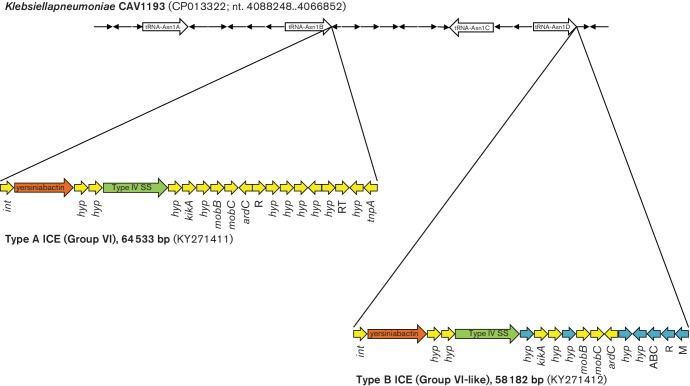
Integrative conjugative elements mobilizing the yersiniabactin cluster. Type A ICE (group VI as defined by Marcoleta *et al*. [32]) identified in strain 48-IT and type B ICE (group VI-like) identified in strain H151440671-UK are drawn indicating their integration sites with respect to the tRNA genes (tRNA Asn1A, 1B, 1C and 1D as described in Marcoleta *et al.* [32]) as detected in the complete genome sequence of strain CAV1193, which does not contain ICEs. Arrows indicate genes and their direction of transcription. Colours indicate clusters encoding the yersiniabactin system (brown), Type IV secretion system (green), hypothetical proteins (blue) and other ICE-associated genes (yellow), respectively. R, restriction; M, methylation; RT, reverse transcriptase.

The two ICEs also showed two different integration sites in their respective *Kp* genomes, the tRNA-Asn1B for type A and tRNA-Asn1D for type B (Fig. 4) [32, 39].

### ST307 fimbriae

A region of approximately 13 kb encoding a π-fimbrial chaperone/usher pathway, comprising the fimbrial subunit, the usher and chaperone proteins, was identified in all ST307 sequenced genomes. The π-fimbriae have been previously described in uropathogenic, piliated *Escherichia coli* [40]. Results of PCR analysis confirmed the presence of this π-fimbrial cluster in all ST307 isolates included in this study (Table 1). Results of genome comparison indicated that this cluster was missing in the ST258 and ST11 genomes, and a blastn search on the entire GenBank nr/nt database identified the cluster in only 15 *Kp* genomes, belonging to different STs (ST147, ST273, ST392, ST86, ST278, ST37, ST941 and ST442).

Six additional fimbriae-encoding clusters and the *mrk* cluster coding for type 3 pili were also identified in all ST307 genomes (Table S4) but these are not unique to the ST307 clone and have been described in many other *Kp* genomes [41].

### ST307 capsular loci and resistance to serum complement

Sequencing of the capsular loci identified the *wzi-173* allele, previously associated with the KN2 capsular type [42, 43] in 20 out of 24 ST307 isolates (Table 1). The four isolates failing to amplify the *wzi* gene included sequenced strain CIV4-IT. In the latter, results of sequence analysis indicated that the capsular cluster was disrupted by an IS*Kpn7* element at the *kpb6* gene and a significant proportion of the genes constituting the cluster were missing. This seems to indicate that the integration of the insertion sequence was followed by a deep rearrangement causing the deletion of approximately 12 ORFs of the *cps*-cluster (ΔCp1 in Fig. S5, panel A).

Besides ST307, *wzi-173* was also identified in two *Kp* isolates in the BIGSdb‐Kp database (http://bigsdb.pasteur.fr/klebsiella/klebsiella.html) belonging to ST1272 (KP-11 and KP-7) isolated in North America from humans. A second complete cluster potentially encoding a different capsular type was identified in all ST307 genomes (Cp2 in Fig. S5 panel B). It was located in a 14 kb region that was part of the 15 discontinuity regions identified by sequence comparison of ST307 with respect to the ST258 NJST258, ST11 HS11286 and ST258 KPN1H1 reference genomes. This cluster showed sequence similarity (93 % nucleotide identity) with capsular clusters previously identified in *Klebsiella quasipneumoniae* strain ATCC 700603 (CP014696.2) and *Enterobacter aerogenes* strain CAV1320 (CP011574). In all the other *Kp* genomes two additional hypothetical proteins are encoded at this site.

**Fig. 5.**
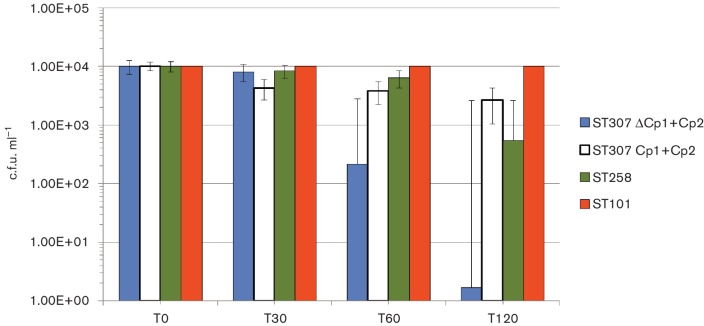
Complement resistance of ST307. Bars represent serum resistance tested using fresh, non-heated human sera obtained from healthy volunteers on ST307 strains 48-IT (blue, representative of strains carrying both Cp1 and Cp2 capsular loci), and CIV4-IT (white, representative of strains carrying ΔCp1 and Cp2 clusters). As comparators strains ST258 (green) and ST101 (orange) were also tested. Colony-forming units were measured immediately after 1 : 3 mixture with sera (T0) and after 30 (T30), 60 (T60) and 120 (T120) min of incubation.

Isolates carrying both Cp1 and Cp2 capsular clusters and those showing the deleted Cp1 cluster were analyzed for complement resistance using a pool of three different fresh human sera from healthy volunteers at a final concentration of 20 %. ST258 and ST101 strains were also tested in the same experiments as internal comparators of the experiments. Results indicated that ST307 isolates endowed with intact Cp1 and Cp2 clusters were more resistant to complement than ST258, but both were more susceptible than ST101. However, IS*Kpn7*-mediated disruption of the Cp1 cluster strongly affected the complement resistance of *K. pneumoniae*, despite the presence of Cp2, showing 2 logs of reduction in c.f.u. in the first hour, and 4 logs of reduction after two hours of incubation with human sera (Fig. 5).

### Other features

Other discontinuity regions were detected by comparing ST307 with the reference ST258 and ST11 genomes. These regions encoded sugar transport via the phosphoenolpyruvate phosphotransferase system, antirestriction proteins, toxin–antitoxin systems, ethanolamine utilization and other functions whose roles in the fitness and virulence of ST307 cannot be predicted (Table S2). Notably, a Type VI secretion system (T6SS) was identified in all ST307 genomes and by blastn in only three other *Kp* genomes (CAV1016-ST45, CP017934; MGH 78578-ST38, CP000647; Kp52.145-ST66, FO834906), and corresponded to the cluster II T6SS, previously described in MGH 78578 [44]. ST307 also carried a cluster for the metabolism of the 4-hydroxyproline that exists in collagen, which most bacteria cannot metabolize [45]. This cluster was detected by blastn in another 17 *Kp* genomes in GenBank with the majority belonging to the ST147 clone.

## Discussion

KPC-*Kp* ST307 is a novel lineage that has potential to become an epidemic or ‘high risk’ clone. Our analysis revealed that ST307 represents a distinctive clonal group and demonstrated that the KPC enzyme was acquired through horizontal transfer of plasmids. In each country of isolation, the most frequent KPC variant on its respective plasmid type (i.e. pKpQIL-KPC-3 and IncN-KPC-2) moved into ST307 [2, 27, 36, 46]. The acquisition of KPC was probably subsequent to that of CTX-M-15 and this event occurred independently in different countries after the spread of ST307, as deduced by the fact that strains had distinct KPC plasmids but related CTX-M-15-carrying plasmids.

We found major characteristics that may provide an advantage to this clone in adaptation to the hospital environment and the human host. Plasmid pKPN-307 is likely to be one of the crucial players in the evolution of this clone. The largest variant of this plasmid identified in this study (type A) carried five putative virulence clusters: the *lac*ZYI operon, the Fec-like iron (III) dicitrate and the glutathione ABC-transport systems, the urea transport system and the cluster for glycogen synthesis. In *E. coli*, glycogen synthesis is regulated by the stress sigma factor RpoS and is considered to be one of the possible adaptive responses to long-term survival and growth in environments outside the host [47]. It can be suggested that plasmid-mediated glycogen synthesis may help ST307 isolates to survive under limited nutrient availability and that the urea transport system may facilitate colonization of the urinary tract by this clone. Urinary tract colonization may also be sustained by the unusual π-fimbria identified in all of our ST307 genomes. This kind of fimbria is characteristic of uropathogenic *E. coli* [40]. ST307 also carries the yersiniabactin siderophore mobilized by an ICE, previously recognized as a relevant and frequent virulence factor in *Kp* [37, 41].

Two different capsular loci were identified in ST307. One is characterized by the *wzi-173* allele, the second cluster is similar to the capsular cluster of genomes of *K. quasipneumoniae* and members of the genus *Enterobacter* and has never been described in *Kp*. Capsules are used by microbes to escape the host immune response and have been associated with biofilm formation, protection from desiccation and contributing to serum survival [48, 49]. Since there were no functional studies on *cps2*, we cannot predict the role of this additional capsular locus in the ST307 genome. However, we showed that capsulated ST307 isolates endowed with the two clusters were more resistant to serum complement than ST258 isolates. Overall, some of the genetic features identified in the ST307 genome despite the lack of a formal functional validation, are interesting and rare and may contribute to increased fitness, persistence and adaptation of this clone to the hospital environment and the human host.

## Data bibliography

1. Villa L, Feudi C, Fortini D, Iacono M, Bonura C, Endimiani A, et al. Complete genome sequence of KPC-3- and CTX-M-15-producing *Klebsiella pneumoniae* sequence type 307. *Genome Announc* 2016; 7:4. pii:e00213-16. NCBI Bioproject PRJNA295649.
2. Villa L, Doumith M. NCBI Bioproject PRJNA354908.
3. Deleo FR, Chen L, Porcella SF, Martens CA, Kobayashi SD, Porter AR, et al. Molecular dissection of the evolution of carbapenem-resistant multilocus sequence type 258 *Klebsiella pneumoniae.* *Proc Natl Acad Sci U S A* 2014; 111:4988–4993. European Nucleotide PRJNA237670.
4. Snitkin ES, Zelazny AM, Thomas PJ, Stock F, Program NCS, Henderson DK, et al. Tracking a hospital outbreak of carbapenem-resistant *Klebsiella pneumoniae* with whole-genome sequencing. *Sci Transl Med* 2012; 4:148ra116. NCBI Bioproject PRJNA224116.
5. Liu P, Li P, Jiang X, Bi D, Xie Y, Tai C, et al. Complete genome sequence of *Klebsiella pneumoniae* subsp. *pneumoniae* HS11286, a multidrug-resistant strain isolated from human sputum *J Bacteriol* 2012; 194:1841-1842. NCBI BioProject PRJNA78789.
6. Sheppard AE, Stoesser N, Sebra R, Kasarskis A, Deikus G, Anson L, et al. Complete genome sequence of KPC-producing *Klebsiella pneumoniae* strain CAV1193. *Genome Announc* 2016; 4:e01649-15. NCBI BioProject PRJNA246471.
7. Villa L, Carattoli A. 2016 GenBank KY271395–KY271415.
8. Zankari E, Hasman H, Cosentino S, Vestergaard M, Rasmussen S, Lund O, et al. Identification of acquired antimicrobial resistance genes. *J Antimicrob Chemother* 2012; 67:2640-2444. Database: https://cge.cbs.dtu.dk//services/data.php.
9. Carattoli A, Zankari E, García-Fernández A, Voldby LM, Lund O, Villa L, et al*. In silico* detection and typing of plasmids using PlasmidFinder and plasmid multilocus sequence typing. *Antimicrob Agents Chemother* 2014; 58:3895-903. Database: https://cge.cbs.dtu.dk//services/data.php.
10. Bialek-Davenet S, Criscuolo A, Ailloud F, Passet V, Jones L, Delannoy-Vieillard AS, et al. Genomic definition of hypervirulent and multidrug resistant *Klebsiella pneumoniae* clonal groups. *Emerg Infect Dis* 2014; 20:1812–1820. European Nucleotide Archive PRJEB6688.

## Supplementary Data

1527 Supplementary File 1
