# Supplementary material for: Diversity, virulence, and antimicrobial resistance of the KPC-producing Klebsiella pneumoniae ST307 clone
Source: Microb Genom. 2017 Apr 26;3(4):e000110. doi: 10.1099/mgen.0.000110 (PMC5506382; doi:10.1099/mgen.0.000110)
Supplement: Supplementary File 1 [file mgen-3-110-s001.pdf]

Table S1. Characteristics and features of the ST307 genomes

Strain	Size (bp)	ORFs	RNAs	Contigs	Accession No.
48-IT	5,682,001	5373	81	112	LKAB00000000
CIV4-IT	5,580,815	5310	129	115	MRBE00000000
KH-43-CO	5,724,434	5472	125	169	MQUU00000000
KL-49-CO	5,680,218	5394	125	140	MQNA00000000
H150820806-UK	5,586,185	5302	81	179	MQUT00000000
H154440769-UK	5,541,345	5246	79	188	MQUZ00000000
H151300628-UK	5,498,686	5207	85	128	MQUW00000000
H151400610-UK	5,485,228	5226	79	211	MQUX00000000
H151400611-UK	5,636,878	5533	85	546	MQUY00000000
H151440672-UK	5,489,845	5223	82	154	MQUV00000000
H155360912-UK	5,439,556	5136	85	130	MQVB00000000
H151440671-UK	5,358,482	5043	86	126	MQVA00000000

Table S2. Major chromosomal differences identified by genome comparison among ST307 48-IT and HS11286, NSJ258_2 and KPNIH1 reference genomes

Contig[a]	Positions[a]	Features	HS11286 ST11 (wzi74)	NJST258_2 ST258 (wzi154)	KPNIH1 ST258 (wzi154)	48IT ST307 (wzi173)
1	756959-821560	ICE, T4SS [b], yersiniabactin	+	-	-	+
1	121459-129485	Cellobiose PTS [c]	+	-	-	+
2	178022-200498	T6SS [b]	-	-	-	+
2	398541-434010	Type 1 Fimbria, RND multidrug efflux	-	+	+	+
3	57266-78773	RND efflux system, acrivinflavin resistance	+	-	+	+
3	175062-197386	Phage ϕ2	+	-	+	+
4	300588-377780	Phage ϕ1	-	-	-	+
4	30798-42976	Capsule 1	-	-	-	+
6	315697-323095	4-hydroxyproline metabolism	-	-	-	+
10	8054-44477	Antirestriction KlcA, YfiZ-YpJF antitoxin-toxin	-	-	-	+
12	1-13623	T1SS[b]	+	-	+	+
12	134263-142016	Sorbitol PTS [c]	-	-	-	+
18	69121-82910	π-fimbrial cluster, urophatogenic	-	-	-	+
18	30161-44180	Capsule 2 Enterobacter	-	-	-	+
28	13267-20969	Ethanolamine utilization	-	-	-	+

[a]: Contig positions refers to the 48-IT genome sequence Acc. No. LKAB00000000

[b]: T4SS:Type IV Secretion System, T6SS: Type VI Secretion System, T1SS: Type I Secretion System

[c]**:** Sugar transport process via the phosphoenolpyruvate phosphotransferase system (PTS)

Table S3. Primers used in this study for PCR amplification of specific ST307 features

Target	Oligo name	Sequence	Amplicon size (bp)	Annealing temperature (°C)
π-fimbrial cluster	Usher_C18Fw	GATCATAGCAACGGTTCGGA	628	57
	Usher_C18Rv	TCAAATGACAGCGCTCCCA		
Capsule 2 Enterobacter	CA_C18Fw	TACCGTTATTCTTGCATCTC	499	55
	CA_C18Rv	CGCGAGATCAAACATACCGG		
Phage ϕ48	C4_Fw	GAAATGGCTTGCTCATGTG	679	52
	C4_Rv	CGGCATTTTCTCACCTGTC		
Glycogen synthesis cluster on pKPN-307	glico-fw	TGATAGGCAATGTTGTG	373	50
	glico-Rv	CCTGGGTTTCCGGCGATCCGC		
Urea transport system on pKPN-307	urtC-fw	CGCCCGCCCACGGCACA	217	60
	urtC-rv	CTCGGCCGACTGATGGTTGCC		
FIB replicon on pKPN-307	FIB-KN fw	GCTGCTCCATCGCCTTACGC	631	57
	FIB-KN Rv	GAAATCAACAGGGAAGAGCAAGG		
FIB replicon on pKpQIL	FIB-KQ fw	GTATCTGGTCTGTTAAGTCG	258	57
	FIB-KQ rv	TCTGCTGGTTATTGGGTGAG		

Table S4. Fimbriae and virulence genes identified in the ST307 genomes

Strain	Fimbriae[a]						*mrk* alleles	*ybt alleles*	*fyuA, irp1, irp2 alleles*
	1040037<br>1040041	160037<br>160044	20008<br>20010	220009<br>220012	610080<br>610083	90118-26<br>90133-37			
**48-IT**	Contig 14	Contig 2	Contig 3	Contig 3	Contig 7	Contig 2	mrkA_12, mrkB-2, mrkD-8, mrkF-4, mrkH-2, mrkI-4, mrkJ-2	ybtA-38, ybtE-5, ybtP-25, ybtQ-6, ybtS-41, ybtU-39, ybtX-11	fyuA-11, irp1-37
**CIV4-IT**	Scaffold 17	Scaffold 14	Scaffold 7	Scaffold 7	Scaffold 0	Scaffold 10	mrkB-2, mrkD-8, mrkF-4, mrkH-2, mrkI-4, mrkJ-2	ybtA-38, ybtE-5, ybtP-25, ybtQ-6, ybtS-41, ybtT-5, ybtU-39, ybtX-11	fyuA-11, irp1-37
**KH-43-CO**	Scaffold 17	Scaffold 13	Scaffold 21	Scaffold 29	Scaffold 0	Scaffold 8	mrkA_12, mrkB-2, mrkD-8, mrkF-4, mrkH-2, mrkI-4, mrkJ-2	neg	neg
**KL-49-CO**	Scaffold 12	Scaffold 18	Scaffold 0	Scaffold 0	Scaffold 1	Scaffold 7	mrkA_12, mrkB-2, mrkD-8, mrkF-4, mrkH-2, mrkI-4, mrkJ-2	neg	neg
**H154440769-UK**	Scaffold 11	Scaffold 0	Scaffold 4	Scaffold 4	Scaffold 6	Scaffold 0	mrkA_12, mrkB-2, mrkD-8, mrkF-4, mrkH-2, mrkI-4, mrkJ-2	neg	neg
**H150820806-UK**	Scaffold 13	Scaffold 0	Scaffold 2	Scaffold 2	Scaffold 5	Scaffold 0	mrkA_12, mrkB-2, mrkD-8, mrkF-4, mrkH-2, mrkI-4, mrkJ-2	neg	neg
**H151300628-UK**	Scaffold 17	Scaffold 1	Scaffold 0	Scaffold 0	Scaffold 6	Scaffold 1	mrkA_12, mrkB-2, mrkD-8, mrkF-4, mrkH-2, mrkI-4, mrkJ-2	ybtA-3, ybtE-9, ybtP-3, ybtQ-4, ybtS-16, ybtT-10, ybtU-3, ybtX-12	fyuA-39, irp1-54, irp2-61
**H151400610-UK**	Scaffold 16	Scaffold 15	Scaffold 46	Scaffold 20	Scaffold 22	Scaffold 3	mrkA_12, mrkB-2, mrkD-8, mrkF-4, mrkH-2, mrkI-4, mrkJ-2	ybtA-3, ybtE-9, ybtP-3, ybtQ-4, ybtS-16, ybtT-10, ybtU-3, ybtX-12	fyuA-39, irp1-54, irp2-61
**H151400611-UK**	Scaffold 36	Scaffolds 323,447,223	Scaffold 99	Scaffold 9	Scaffold 87	Scaffold 179-212-28	mrkB-2, mrkD-8, mrkF-4, mrkH-2, mrkI-4, mrkJ-2	ybtP-3, ybtQ-4, ybtS-16, ybtT-10, ybtU-3, ybtX-12	fyuA-39
**H151440672-UK**	Scaffold 4	Scaffold 17	Scaffold 1	Scaffold 2	Scaffold 20	Scaffold 5	mrkA_12, mrkB-2, mrkD-8, mrkF-4, mrkH-2, mrkI-4, mrkJ-2	ybtA-3, ybtE-9, ybtP-3, ybtQ-4, ybtS-16, ybtT-10, ybtU-3, ybtX-12	fyuA-39, irp1-54, irp2-61
**H15536091201-IT**	Scaffold 11	Scaffold 2	Scaffold 4	Scaffold 4	Scaffold 1	Scaffold 2	mrkA_12, mrkB-2, mrkD-8, mrkF-4, mrkH-2, mrkI-4, mrkJ-2	ybtA-3, ybtE-9, ybtP-3, ybtQ-4, ybtS-16, ybtT-10, ybtU-3, ybtX-12	fyuA-39, irp1-54, irp2-61
**H151440671-UK**	Scaffold 11	Scaffold 13	Scaffold 2	Scaffold 2	Scaffold 12	Scaffold 3	mrkA_12, mrkB-2, mrkD-8, mrkF-4, mrkH-2, mrkI-4, mrkJ-2	ybtA-3, ybtE-9, ybtP-3, ybtQ-4, ybtS-16, ybtT-10, ybtU-3, ybtX-12	fyuA-39, irp1-54, irp2-61

[a] All usher loci included between the two numbers were identified in the respective scaffolds of assembled genomes (i.e. Usher_1040037, Usher_1040038, Usher_1040039, Usher_1040040 and Usher_1040041 are indicated as 1040037-1040041 in Table) . Usher loci numbers refer to nomenclature adopted at the BIGSdb-Kp database (http://bigsdb.pasteur.fr/)

Figure S1. Comparative analysis among ST307 genomes using the *K. pneumoniae* 48-IT strain as reference genome

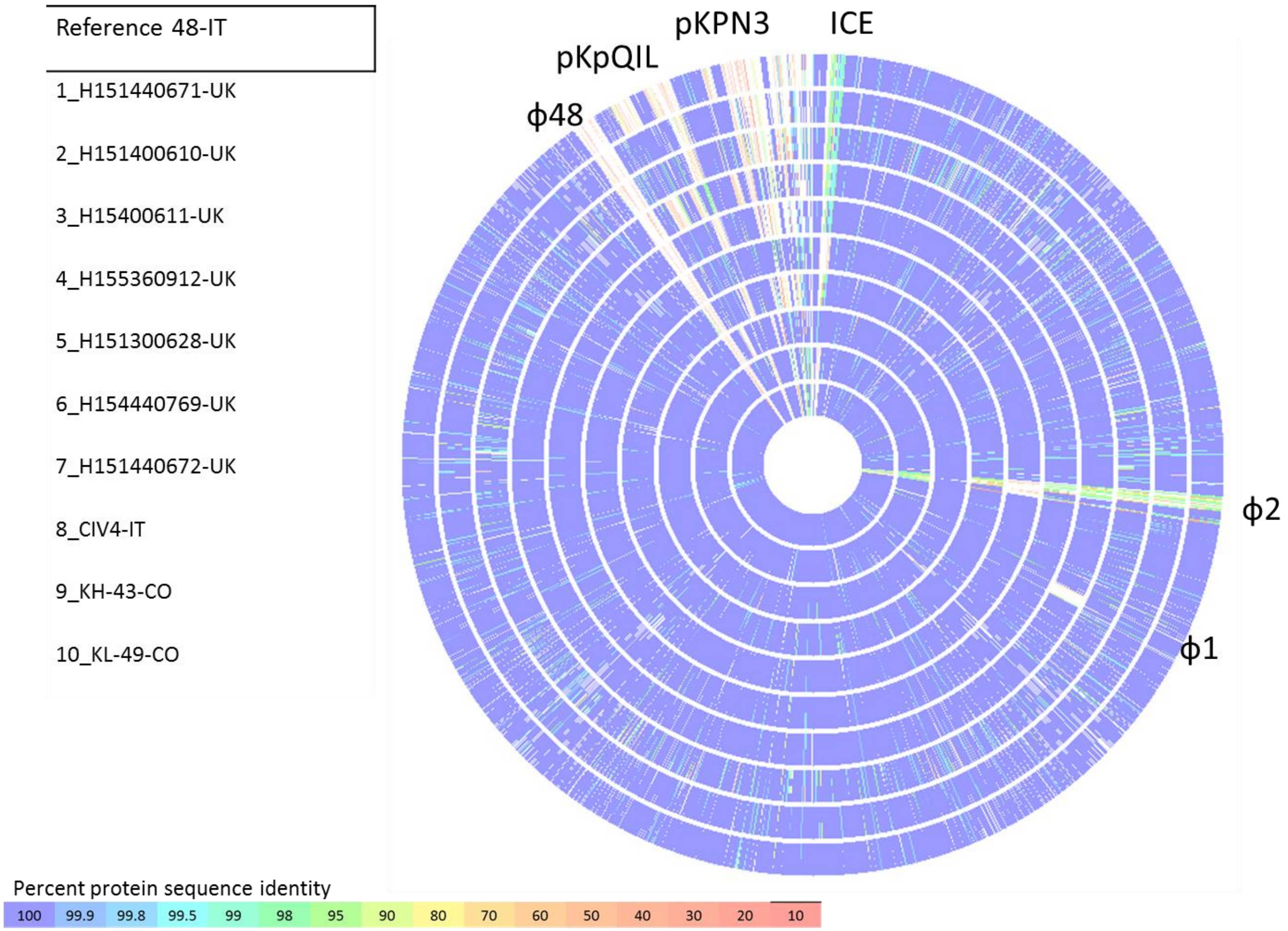

The comparison was obtained uploading the ST307 annotated genomes at the RAST server (http://rast.nmpdr.org/seedviewer.cgi) by Blast P analysis of CDS, visualized by the Seed Viewer version 2.0. The major differences among the strains of the ST307 clone consisted in phages (ϕ48), prophages (ϕ1 and ϕ2), plasmids (pKPN3 variants and pKpQIL presence or absence) and ICE typeA and type B content.

Figure S2. Comparative analysis of ST307 genomes *vs Klebsiella pneumoniae* complete reference genomes

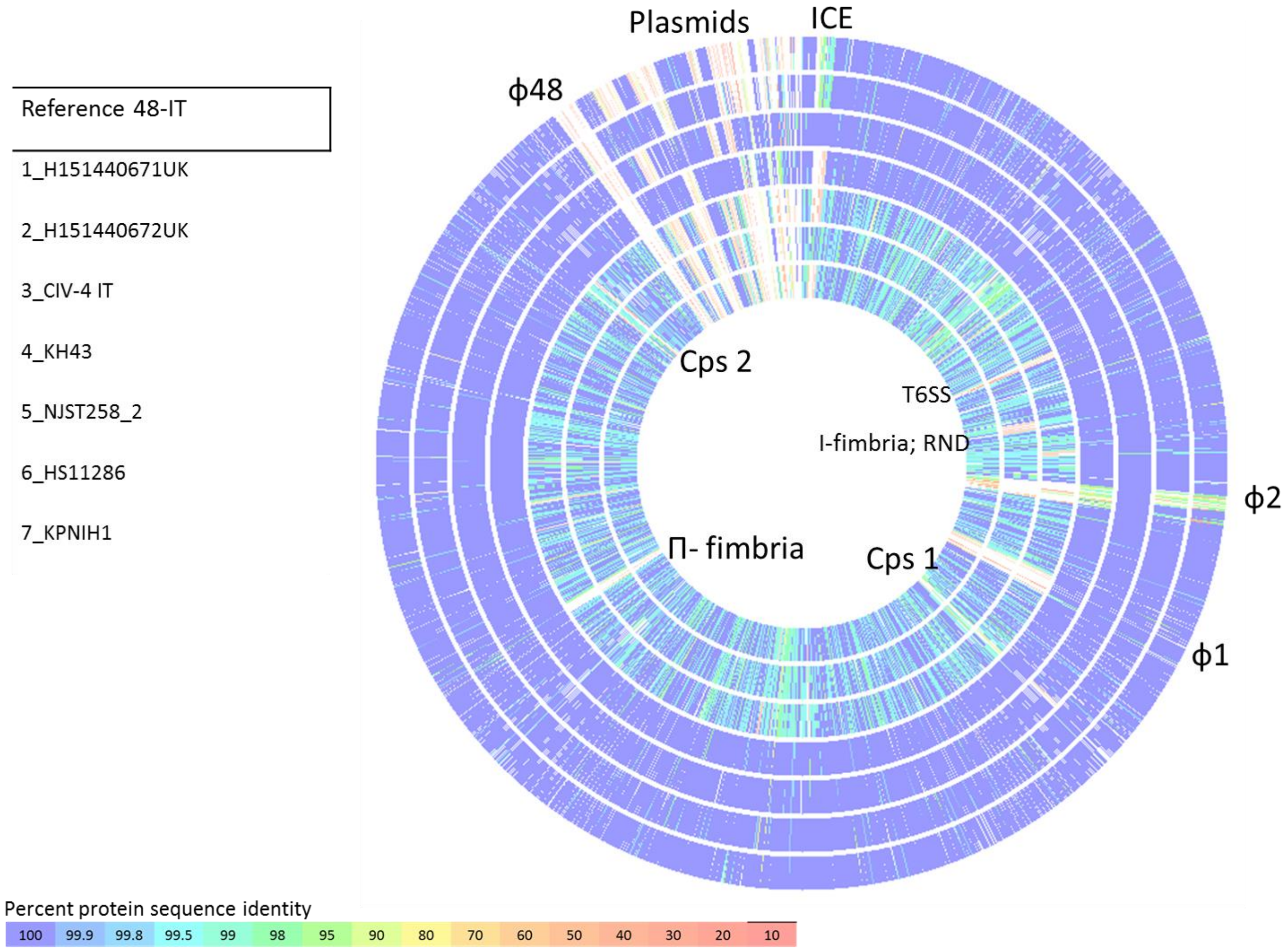

The comparison was obtained uploading 3 prototypic ST307 genomes and the *K. pneumoniae* NJST258_2 (NZ_CP006918), KPNIH1 (NZ_ CP008827) and HS11286 (CP003200), reference genomes at the RAST server (http://rast.nmpdr.org/seedviewer.cgi) by Blast P analysis of CDS, visualized by the Seed Viewer version 2.0. The major differences identified in this comparison are listed in Table S2.

Figure S3. Alignment by MAUVE of ST307 prototypic genomes *vs* complete reference genomes

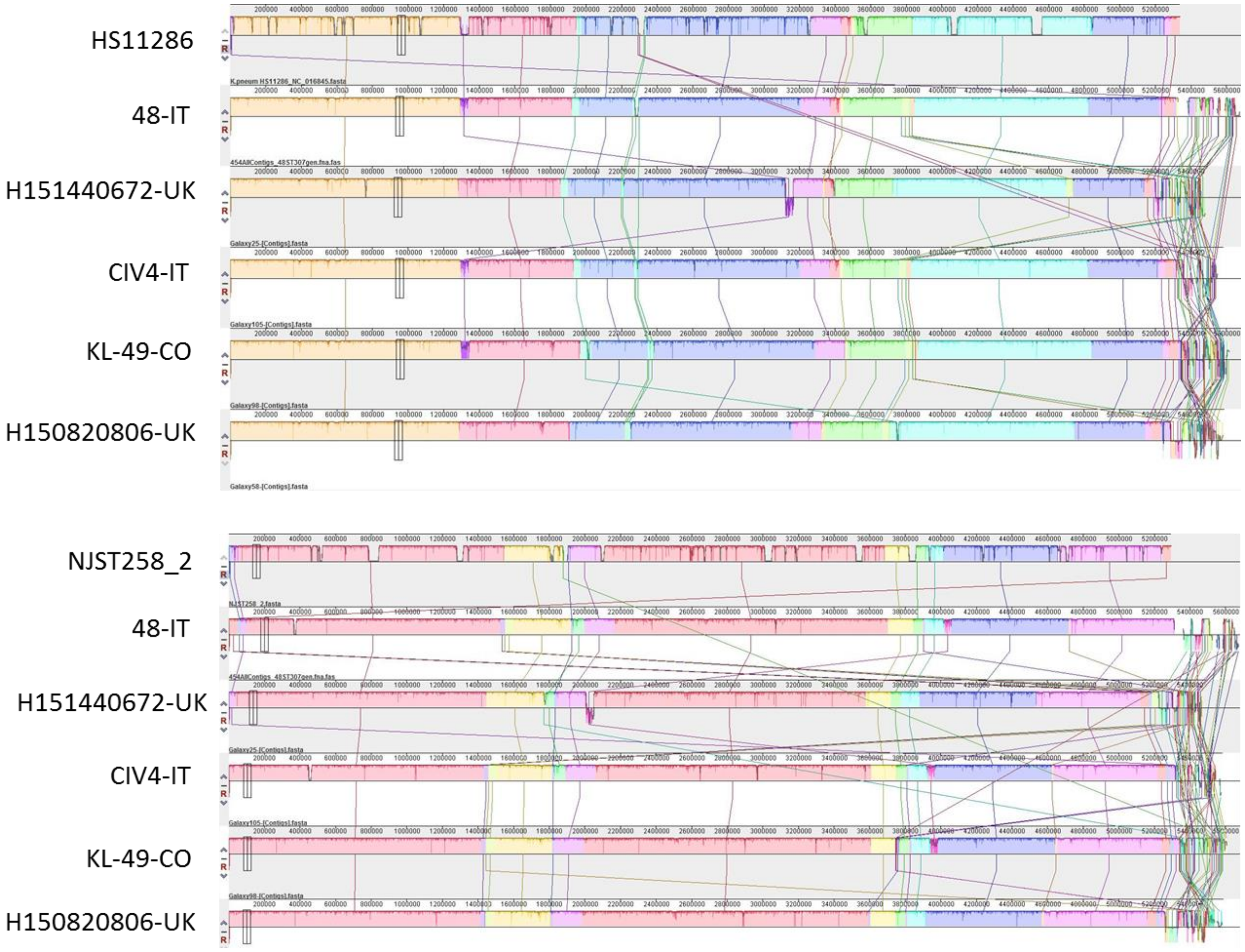

ST307 genomes were ordered and aligned using the MAUVE comparison tool against the *K. pneumoniae* NJST258_2 (NZ_CP006918), and HS11286 (CP003200) reference genomes.

Figure S4. Prophages identified in the ST307 genomes and ϕ48

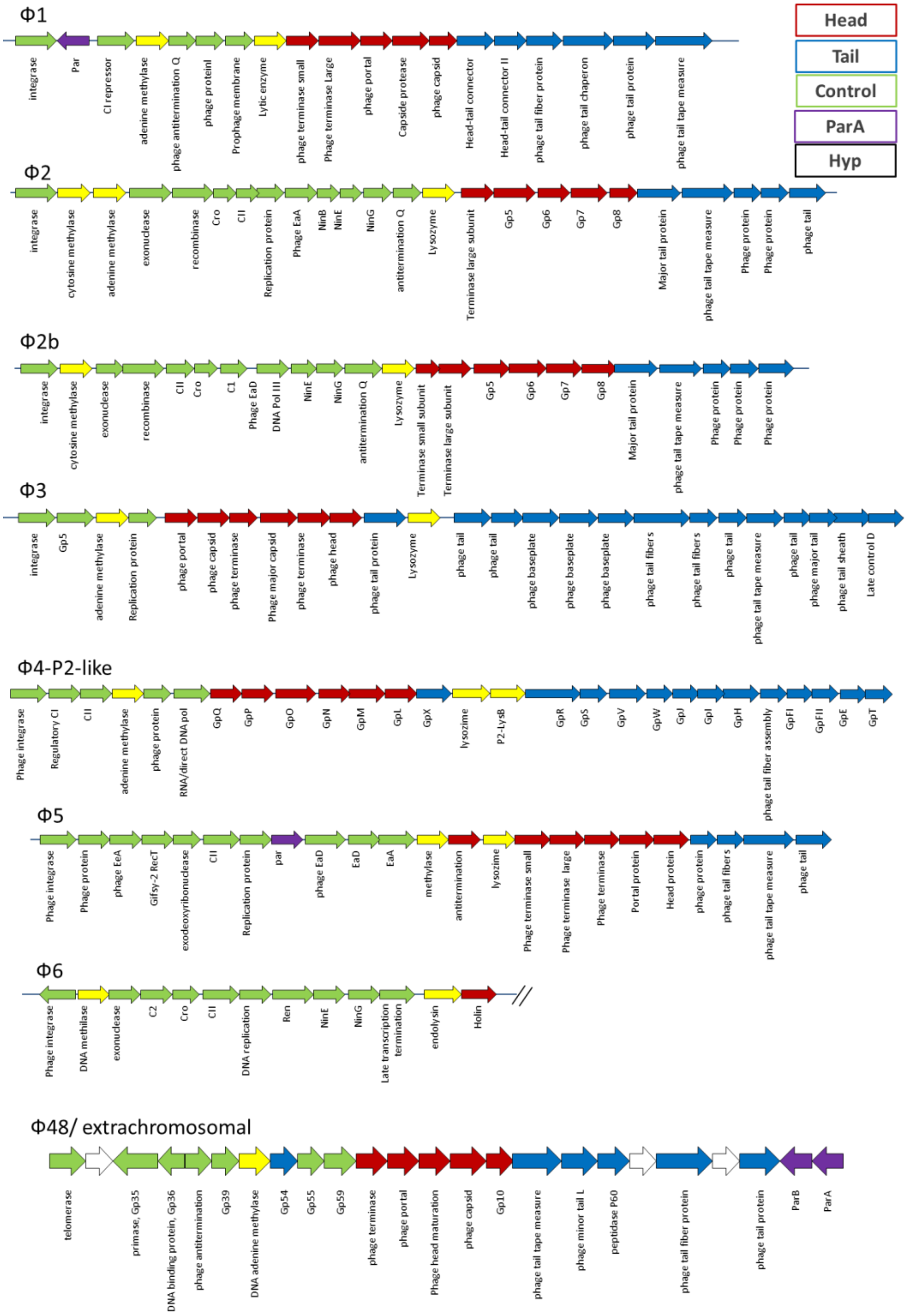

Arrows indicate phage genes and their direction of transcription.Colours indicate clusters encoding replication control (green), capside assembly (red), tail assembly (blue). Partitioning and lysozyme encoding genes are indicated by violet and yellow arrows, respectively

Figure S5. Capsule gene cluster in ST307, ST258 and ST101

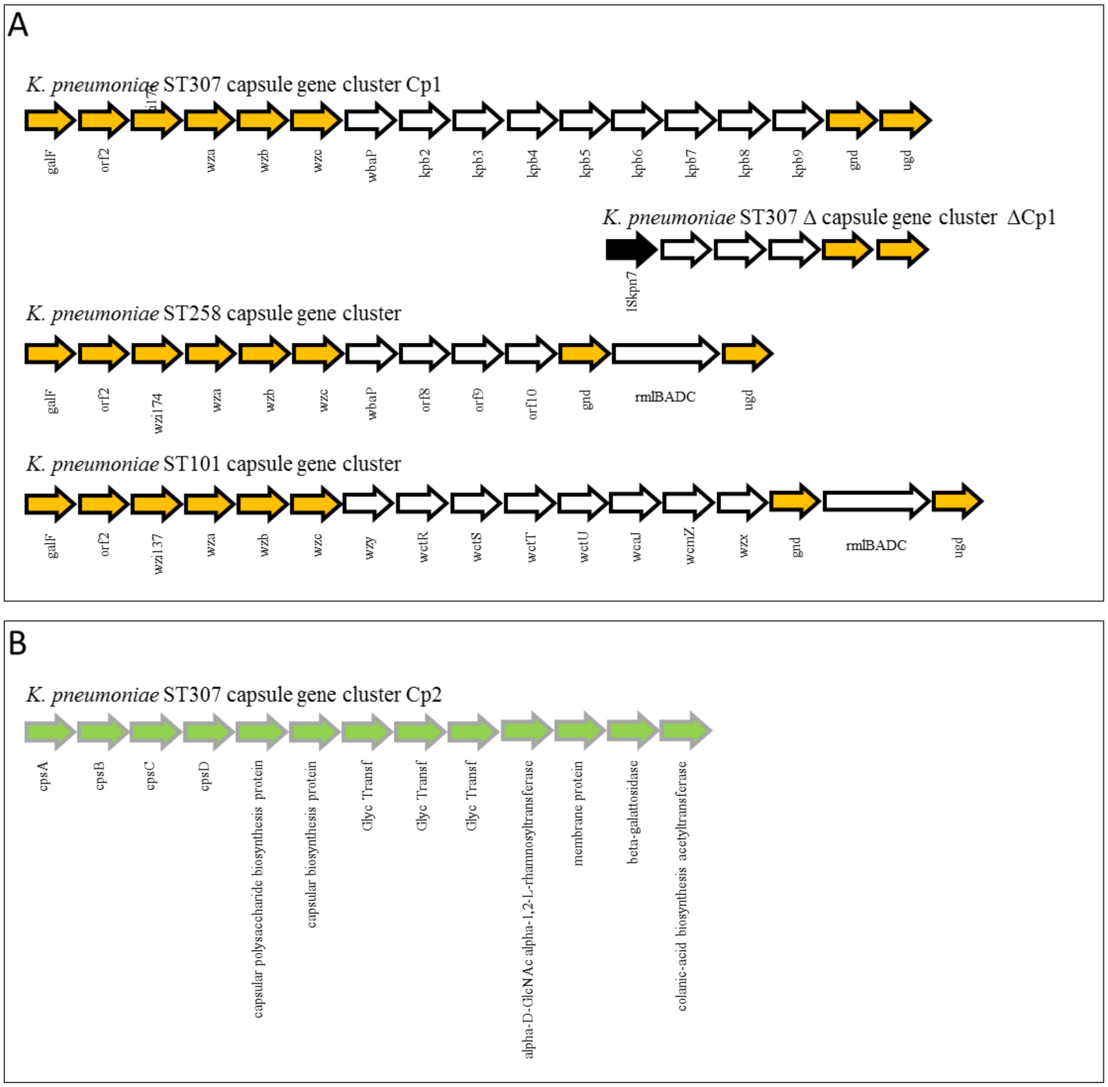

Gene clusters of the two capsular clusters Cp1 (panel A) and Cp2 (panel B) identified in ST307. The intact Cp1 and IS*Kpn7* deleted ΔCp1 are drawn in comparison to the capsular loci of ST258 and ST101 strains. Arrows indicate genes and their direction of transcription. Yellow arrows indicate genes that are common, white arrows those that are different among ST307, ST258 and ST101 clusters. The second capsular cluster Cp2 is indicated by green arrows
